# A new method to improve the prediction of the celestial pole offsets

**DOI:** 10.1038/s41598-018-32082-1

**Published:** 2018-09-14

**Authors:** Santiago Belda, José M. Ferrándiz, Robert Heinkelmann, Harald Schuh

**Affiliations:** 1 Department of Applied Mathematics, University of Alicante, Carretera San Vicente del Raspeig s/n, San Vicente del Raspeig, E-03690 Alicante, Spain; 2 Helmholtz Centre Potsdam, German Research Centre for Geosciences (GFZ), Telegrafenberg, A17, D-14473 Potsdam, Germany

## Abstract

Knowledge of the Earth’s changing rotation is fundamental to positioning objects in space and on the planet. Nowadays, the Earth’s orientation in space is expressed by five Earth Orientation Parameters (EOP). Many applications in astronomy, geosciences, and space missions require accurate EOP predictions. Operational predictions are released daily by the Rapid Service/Prediction Centre of the International Earth Rotation and Reference Systems Service (IERS). The prediction procedures and performances differ for the three EOP classes: polar motion, rotation angle (UT1-UTC), and the two celestial pole offsets (CPO), dX and dY. The IERS Annual Report 2016 shows Rapid Service CPO predictions errors with respect to IERS 08 C04 observations in 2016 ranging from 120 to 140 μas in 40 days for dX, and 100–160 μas for dY. We test a new method for the CPO prediction based on the recent availability of sophisticated empirical models for the Free Core Nutation, a main component of the CPO variations. We found it allows predicting both CPO with error estimates for the period 2000–2016 lower than the 2016 Rapid Service products, reaching about 85 μas after 40 days and near 90 μas after a year. These results would represent a 35–40% improvement.

## Introduction

The Earth’s orientation in space can be expressed through three independent angles (e.g. the Euler angles). However, five Earth Orientation Parameters (EOP) are preferred since the advent of modern space geodesy techniques such as Global Navigation Satellite Systems (GNSS)^1,2^, Very Long Baseline Interferometry (VLBI)^3–6^, Satellite Laser Ranging (SLR)^7^, and Doppler Orbitography and Radiopositioning Integrated by Satellite (DORIS)^8^. The EOP provide the rotation from a Geocentric Terrestrial Reference System to a Geocentric Celestial Reference System as a function of time, and thus are basic for all positioning and navigation systems and must be monitored as accurately as possible. The Earth Orientation Centre (EOC) of the International Earth Rotation and Reference Systems Service (IERS) obtains and releases a solution with daily EOP values, named C04, which comprise the terrestrial pole coordinates (x_p_, y_p_), the difference UT1-UTC, and the Celestial Pole Offsets (CPO), together with their respective formal errors. The CPO are usually expressed as the deviations dX, dY, between the observed Celestial Intermediate Pole (CIP) and the conventional position of the CIP deduced from the conventional IAU2000A nutation^9^ and IAU2006 precession^10,11^ models, first adopted by Resolutions of the International Astronomical Union (IAU) and then endorsed too by the International Union of Geodesy and Geophysics (IUGG). Precise definitions of the main and auxiliary parameters and frames can be found in^12–14^, for instance.

VLBI is the only technique capable of determining the CPO values with the accuracy currently required for most applications. Roughly speaking, most of the operational CPO determinations performed by the various IERS Analysis Centres (AC) affiliated to the International VLBI Service for Geodesy and Astrometry (IVS^15^) show a level of angular accuracy corresponding to a few millimetres on the Earth’s surface. However, the actual accuracy of any EOP must be inferred from differences among individual and combined solutions and formal errors. Regarding the CPO, the IERS Annual Reports (AR) are a main source for their accuracy assessment. According to the last one published, the AR2016^16^, the estimated accuracies of the contributions to the IERS Rapid Service final products range from 60 to 190 μas for dX and dY (Table 1 on p. 90). Those figures indicate the level of agreement between the solutions computed by the IVS and its main AC; the EOP series of the IERS Rapid Service/Prediction Centre (RS/PC) are not very different from a former uncertainty estimation of about 80–90 μas in average^17^. Those figures seem to be lowering slowly in the last years. According to the estimates for different time periods that appear in the EOC section of the AR2016 (Table 1, p. 86), the standard deviations between the IVS and the EOP 08 C04 solutions^18^ are 56 μas for dX and 74 μas for dY in 2010–2015. For the newer series EOP 14 C04^19,20^ those numbers decrease up to 54 μas for dX and 45 μas for dY in the same period (see Table 2, p. 86, in^16^). Other studies comparing various solutions found differences of some tens of μas among them^21–24^. Those inconsistencies in the CPO determination are usually attributed to differences of software, processing strategies, or standards.

The aforementioned IERS RS/PC, hosted by the U.S. Naval Observatory (USNO), is in charge of predicting the three Earth rotation parameters (ERP), namely x_p_, y_p_, and UT1-UTC, and the CPO as well. The daily EOP combination and prediction solution (files named *finals.daily* or *finals2000A.daily*) is produced at approximately 17:05 UTC each day, and includes predictions up to 90 days in the future. The predictions of the ERP start on the same day of the aforesaid release. However, the CPO predictions often have latency larger than two weeks, because of the delay in the availability of accurate VLBI solutions. The weekly version (*IERS Bulletin A*) is released on every Thursday at 17:00 UTC with predictions of x_p_, y_p_, and UT1-UTC, for up to 360 days into future. However, the CPO predictions are provided with a delay of about one week between the publication date and the last available date with VLBI estimated offsets. These products can be currently accessed from the server at http://maia.usno.navy.mil.

Accurate EOP predictions are needed e.g. in the process of tracking and navigation of interplanetary spacecraft missions and for laser ranging to satellites and to the Moon. In addition, other applications related to astronomy, geodesy, communication and time keeping could benefit from these rigorous estimates. It is important to mention that there is a broad variety of prediction methods which provide different accuracies at different time intervals and prediction lengths, i.e. a method which is the best for short-time prediction may not be such for long-time prediction, and vice versa. Besides, optimally specific methods would be used depending on the EOP groups: ERP and CPO.

The ERP prediction is performed by means of the development and application of algorithms such as the least squares extrapolation (LS) of harmonic model and autoregressive (AR) prediction^25–28^ spectral analysis and least squares extrapolation^29,30^, artificial neural networks (ANN)^31,32^, least squares collocation (LSC)^33^, wavelet decomposition and auto-covariance prediction^34^, Kalman filter forecasts^35,36^ and fuzzy inference system^37^, among others.

According to the description given in the IERS AR2016, “the predictions of celestial pole offsets (both dX/dY and dψ/dε representations) are produced through the use of the KSV1996 model (McCarthy, 1996)^38^”, a semi-analytical model has been used since about 20 years. The last paragraph of each Bulletin A includes a link to the subroutine ceppred.f, recommended to predict the CPO that does not make use of any observations of the CPO (http://www.usno.navy.mil/USNO/earth-orientation/software/aux/ceppred.f). Let us comment that no numerical theory (e.g. ERA solution by Krasinsky^39^ & Krasinsky and Vasilyev^40^) has been able to produce good and stable solutions for the precession/nutation of the non-rigid Earth, unlike other problems like planetary orbits. Although ERA gets good accuracy along the years used to fit the theory, the accuracy decreases quickly when the numerical integration is extrapolated beyond that interval^41^, rendering that numerical solution inappropriate for predictions.

Consideration of the Free Core Nutation (FCN) signal is necessary to improve the modelling of the CPO, since it is the major source of inaccuracy or unexplained time variability with respect to the current IAU2000 nutation theory^9^; the weighted RMS (wrms) of the deviations between observations and IAU2000A model is roughly of the order of 200 µas. FCN is mainly excited by angular momentum exchanges among the mantle and the Earth’s fluids^42–44^ and VLBI is the only technique capable of accurately determining this signal. It has a long retrograde period of about 430 mean solar days (with average amplitude of about 100 µas) relative to the inertial frame^45^, or a period slightly shorter than one day in the retrograde-diurnal band when referred to the rotating terrestrial frame. Nowadays, different empirical FCN models, derived by procedures with various levels of complexity^46–48^ are available. The IERS conventions (2010)^49^ recommend the model of Lambert^50^, which was developed by fitting the FCN amplitude on a two-year interval running forward by steps of one year.

The FCN cannot be known accurately without observations, but some questions arise:

1. Can we make a reasonably accurate prediction of the FCN signal before observing it, taking advantage of new, more sophisticated models?
2. Can we benefit from FCN prediction that improve the current CPO predictions? And,
3. What is the gain in terms of root mean square (RMS)? Does it demand a new prediction procedure?

Following these aforesaid questions and given that the variations of the FCN signal are moderate and seldom abrupt, in this paper we examine whether the availability of a new empirical FCN model^48^, which admits frequent updating, can be exploited to make reasonably accurate predictions of the FCN signal and CPO.

## Methods

### Prediction model proposed by the authors

The main source of variance of the CPO series, seen as deviations from the conventional IAU2000/2006 model, is the FCN signal. The wrms of the CPO series can be significantly reduced by subtracting an accurate FCN model, and therefore it is conceivable that the variance of the CPO predictions may be reduced using a suitable FCN prediction. However, FCN models must be obtained empirically a posteriori, from already determined CPO. Therefore, the possibility of predicting or forecasting FCN is limited, since our knowledge of its geophysical excitation is insufficient. On the other hand, the FCN amplitude is small and exhibits only slow variations. In that way, we may expect that future FCN amplitudes can be predicted from past amplitudes for a longer time interval than other signals, e.g. polar motion, while keeping a reasonable accuracy, as suggested by preliminary tests^51^. The aforesaid idea has been developed by using our current empirical FCN model of Belda *et al*.^48^, hereafter called “B16”. Here we will test if this model can be the basis for a new CPO prediction algorithm.

An alternative procedure may rely on Malkin’s (2013) FCN model^47^, whose features are quite similar (see Table 2 in^48^). It is important to note that these models were estimated together with two constant corrections (X_0_, Y_0_) to CPO in order to avoid a contamination of the FCN signal determination. This reduces the low frequency signals coming from systematic errors in the IAU 2006 precession and IAU2000A nutation models, among other potential factors. Lambert’s FCN model^50^ seems less appropriate for that purpose since it is updated only once a year. FCN could be also recovered from CPO series by numerical integration of certain simplified equations of motion and then to fit suitable parameters^52^. Nevertheless, this procedure is more complex and does not provide either better accuracy or the usual quasi-harmonic representation of FCN. Therefore, we understand that using angular momentum approaches instead of empirical FCN modelling is not the first option for accurate, robust, and simple FCN and subsequent CPO prediction. Other distinct procedures, using e.g. Gaussian smoothing of the IERS EOP series, have been developed^53^, but are not based on FCN prediction.

### Data set

**Figure 1:**
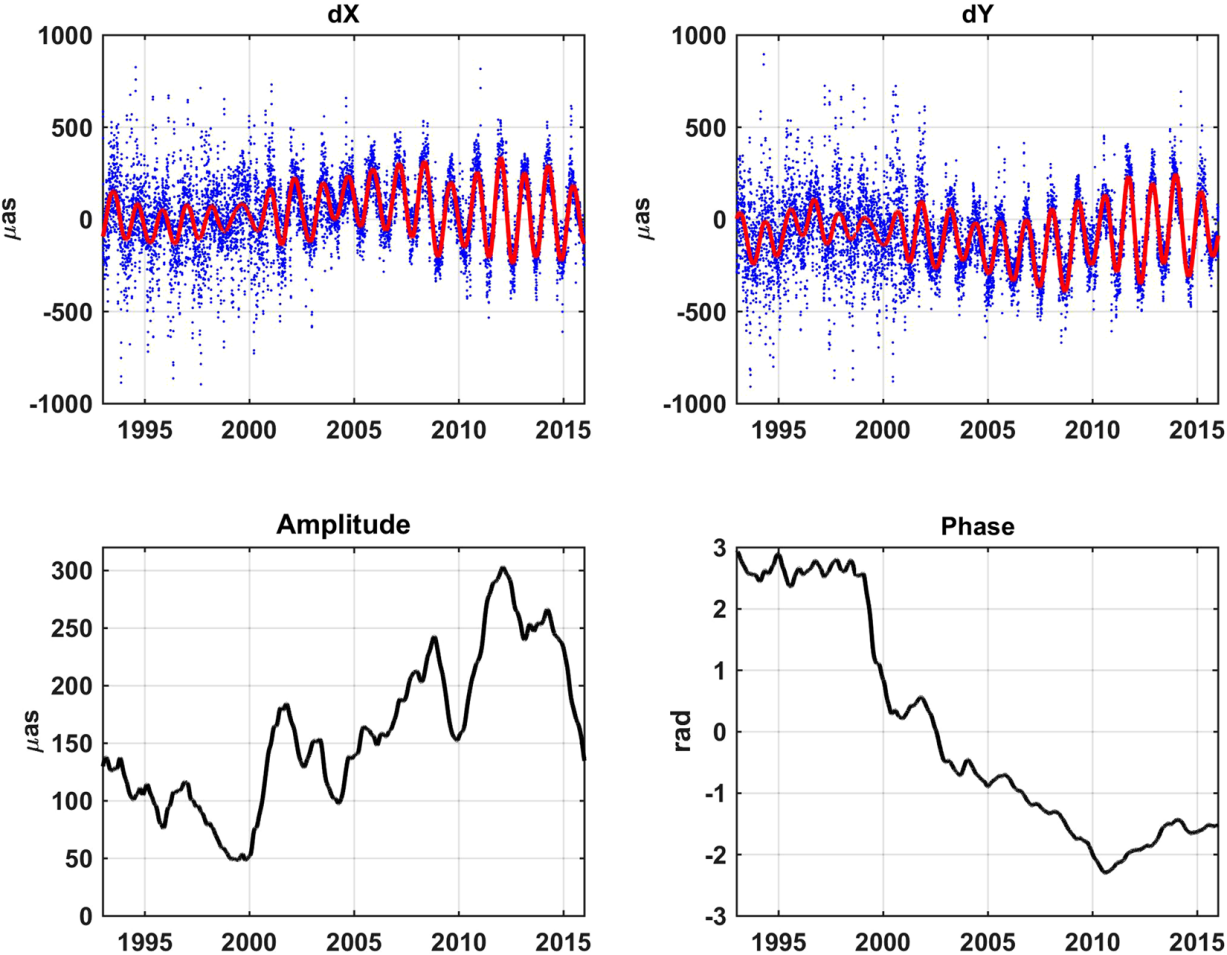
Top: Celestial Pole Offsets (CPO, dX & dY) taken from IERS 08 C04 (blue dots) and FCN model B16 (red line). Bottom: Amplitude and phase variations of the FCN.

The basic data are the conventional IERS EOP 08 C04 CPO series^18^, which are processed with respect to the IAU 2006/2000A precession-nutation model and are consistent with the ITRF2008^54^. Let us notice that the 08 C04 solution has been already replaced by the EOP 14 C04^19^, but all the information on CPO prediction available in the last IERS ARs is still referred to the former 08 C04 series, and therefore we must use the same EOP solution to be able to perform comparisons. The basic data for computing the FCN and thus the CPO predictions is the B16 FCN model. The predictions are compared to the corresponding CPO of the conventional EOP 08 C04 series. The CPO taken for the IERS EOP 08 C04 series (blue dots), the corresponding values of the FCN contributions to the CPO derived from the daily amplitudes provided by the B16 model (red line) as well as its amplitude and phase variations referred to the midpoint of the sliding 400-day window are shown in Fig. 1 with the sampling interval of one day along the period 1994–2016. Let us note that the FCN amplitude showed a general long-time decrease before 1999. It subsequently grew until 2011 and then seemed to decrease again. On the other hand, a similar FCN phase behaviour can also be observed, i.e. the long-time FCN phase drift changed suddenly in 1998–1999 and seemed to change again at the epoch immediately preceding 2011. Consequently, our prediction analysis starts after this big FCN change (2000.0) up to 2016.0, which approximately corresponds to the most precise VLBI-derived Earth orientation parameters (EOP) as compared with earlier observations^47,55^. To validate our CPO predictions, we include an error analysis of the differences between the predictions and the final results obtained from observational data.

## Results

### Capabilities of B16 model for the FCN prediction

First, let us recall that FCN amplitudes of the B16 model are estimated at a daily rate, by using a sliding window 400 days wide and advancing forward by one-day steps. The FCN frequency is fixed and B16 is fitted by weighted least squares from a pre-existing CPO solution, in this case EOP 08 C04. The updating of early methods, like the pioneering Herring’s^56^ or Lambert’s^50^ happens less often, about once a year. The latter model has a two-year fitting window and each tabulated value is assigned to the mid-point of the interval. The last reported amplitude is the basis to extrapolate the FCN model until the next one is released. Then, the amplitude of that last year is changed to a piece-wise linear function joining the last two nodes. Notice that “extrapolation” means here that the CPO are available one year beyond the last tabulated amplitude date, but updating the FCN amplitude requires one year more.

**Figure 2:**
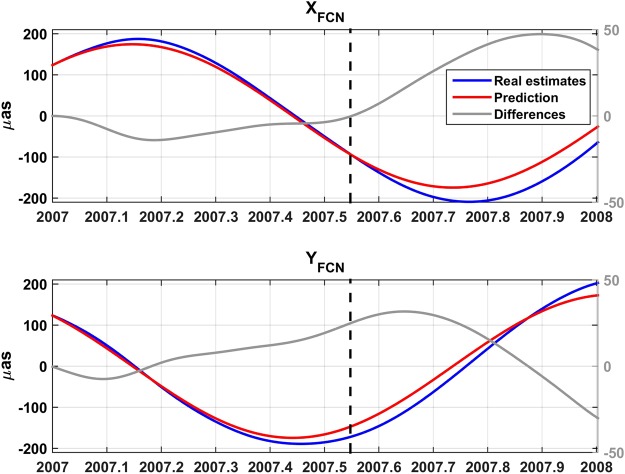
Comparison between the FCN signal given by the B16 model (blue line) and the predicted FCN computed from the sole first day amplitude (red line), with their scales displayed on the left axes. The differences between both curves are shown in grey; notice that its scale, ticketed in grey on the right, is amplified four times. The meaning of the dashed vertical is explained in the text.

To investigate the ability of B16 to predict the FCN signal, we start with a simple test inspired in our comments on the FCN model recommended in the IERS Conventions (2010). We consider some years in which definitive EOP solutions are available. Firstly, we focus on a certain date, concretely at epoch 2007.0, and compute the FCN along a whole year using the FCN amplitude which was estimated from the 08 C04 CPO values of the 200 days before and after the aforesaid epoch. The results for X_FCN_, Y_FCN_, are the red curves displayed in Fig. 2 with the label “Prediction”. Notice that the first 200 days were used to obtain the FCN amplitude in 2007.0, since our tabulated (daily) values are also at the centre of each fitting window; therefore, the prediction in the first interval corresponds to the extrapolation of Lambert’s model mentioned in the previous paragraph. After those first 200 days, the kind of prediction or extrapolation is different, since the corresponding CPO values did not take part in the computation of the 2007.0 FCN amplitude. A dashed vertical line marking the elapsed 200 days, at nearly 2007.55, highlights that difference of meaning. Curves depicted in blue and labelled “Real estimates” correspond to the X_FCN_, Y_FCN_, daily values provided by B16. The differences between the two curves give the error of computing FCN not with its daily B16 amplitude but a fixed one. Until the dashed vertical, the error would correspond to the extrapolation error of e.g. Lambert’s model after the last amplitude release; after that line its meaning changes as explained. They are shown as grey curves, but notice the vertical scale is amplified four times with respect to the blue and red lines. It is clear that the extrapolation or prediction error is lower in the first stretch, but even in the second it does not reach 50 µas. For the sake of shortness, we will use sometimes the terms “partial” or “complete” predictions to distinguish both cases.

**Figure 3:**
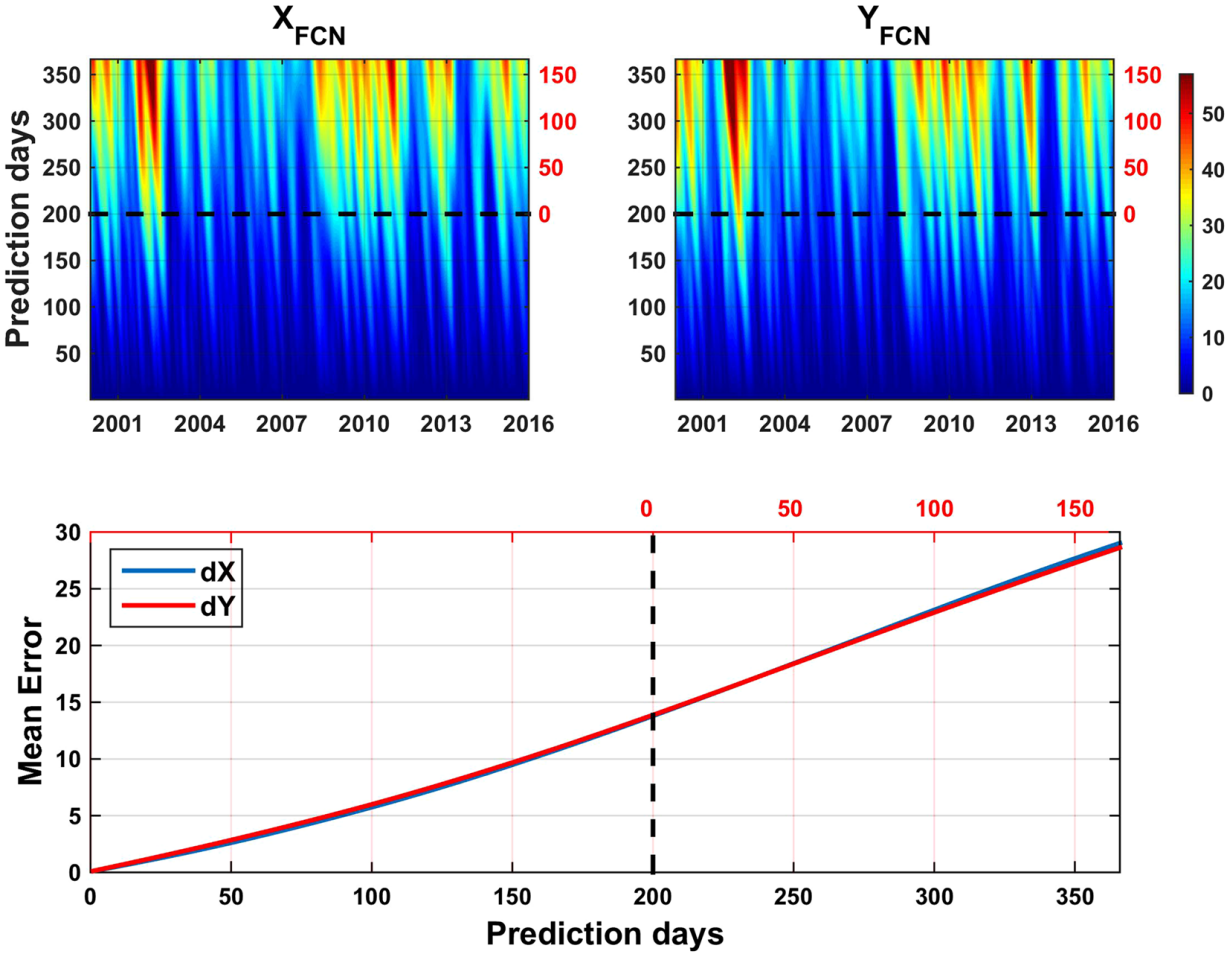
Daily RMS of the differences (top), along with the mean prediction errors (bottom) between the B16 modelled FCN and the FCN predicted like in Fig. 2, for the full period 2000–2016. Red color figures of the axes correspond to days since the beginning of the complete prediction, see the text for explanation. Sampling interval: 1 day. Units: µas.

The slight differences of the pair of curves for each CPO component (grey line, Fig. 2) suggest the proposed methodology may be valid. To raise that to a conclusion, we need much more than the results for a specific date. Therefore, we repeat the same experiment starting each day of the period 2000.0 to 2016.0, and predicting one year of FCN since it. The differences between the actual B16 FCN values and those predicted as before (alike grey curves in Fig. 2) are shown in Fig. 3. The upper part shows the RMS of the differences. Dark blue colors are dominant in the “partial” prediction days (below 200 days line), while RMS seldom reaches 40–50 µas in the “complete” prediction period (above 200 days line). The lower plot displays the mean values for each horizontal line of the upper plots. Despite the variability of the FCN amplitude and phase, Fig. 3 clearly evidences high accuracy and stability for ultra-short, short-, mid- and long-term predictions (mean prediction error of roughly 30 µas after a year). For easiest reference, the number of days reckoned from the 200^th^ day since the date where each prediction period starts is displayed in red color in the right or upper part of the top or bottom plots, respectively.

### CPO prediction

**Figure 4:**
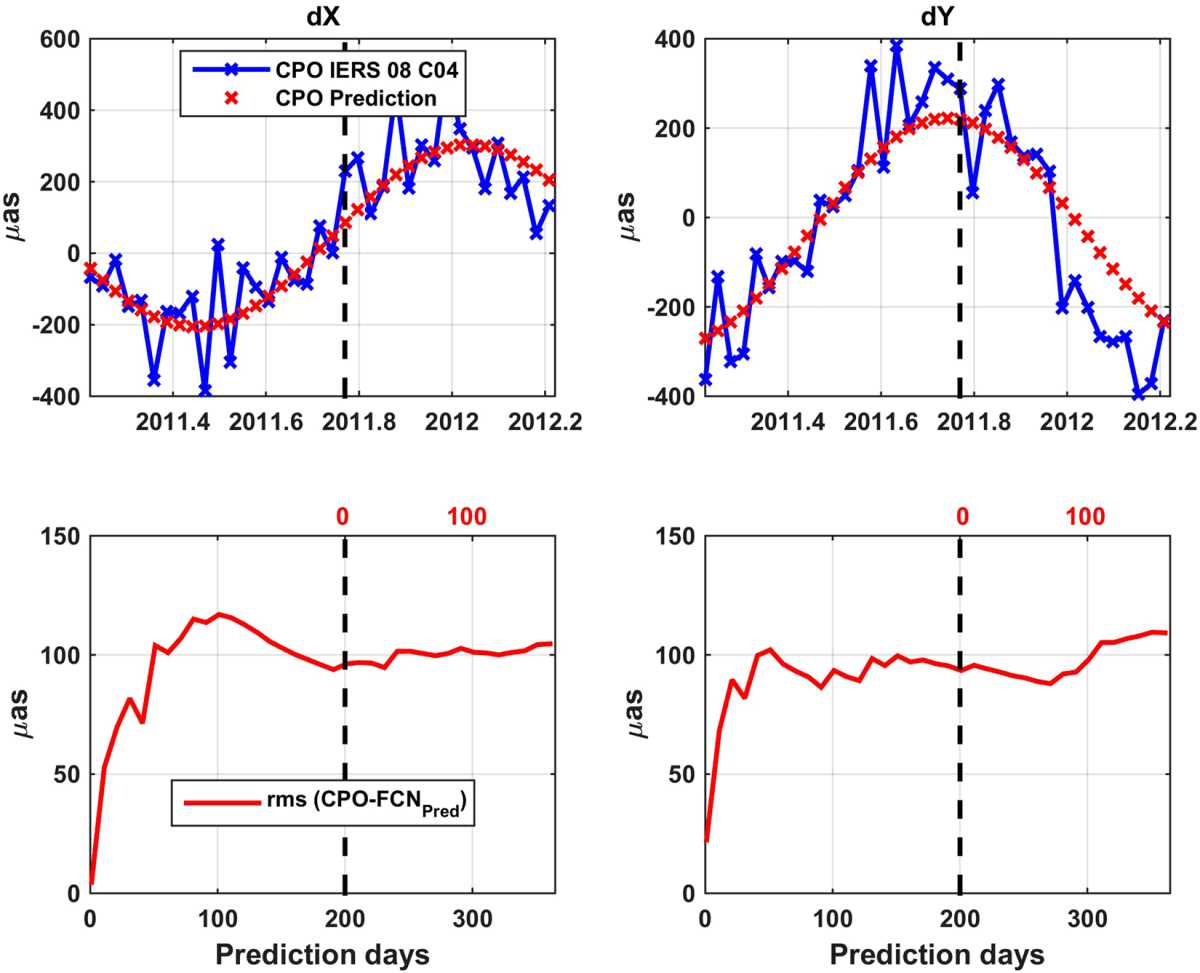
Top: “IERS 08 C04” CPO series (blue) vs. CPO predictions (red) estimated from past amplitudes of the FCN model B16. Bottom: RMS of the differences between the aforesaid series. Black and red scales of time and 200 days line as in Figs 2 and 3. Time frame of the prediction: 2011.2–2012.2.

After assessing the ability of our B16 FCN model to extrapolate the FCN signal, a second study addressed the differences between the CPO values predicted with the help of B16 and the conventional determinations IERS EOP 08 C04 CPO time series. The procedure for the CPO prediction followed in this subsection is simple: Daily CPO values are predicted by extrapolating the FCN model with the amplitude derived from the 08 C04 CPO values of the 400 days preceding the prediction epoch. Keeping the same scheme as before for presenting the results, i.e. local and global analysis, we first consider a specific time span (2011.2–2012.2) with the results depicted in Fig. 4. The generation of the FCN components is similar to that of Fig. 2. The comparison between the CPO predictions (red color) against the IERS 08 C04 values, which are taken as exact disregarding their (unknown) actual errors (blue color) shows a maximum RMS of the deviations between each pair of series of about 100 µas, which is kept almost steady between 50 and 365 days (Fig. 4, bottom). However, there is a rapid increase of the errors when going from the ultra-short, to short- and mid-term predictions. We suspect that the behavior could be improved taking also into account geophysical excitation functions and forecasts thereof ^57^.

**Figure 5:**
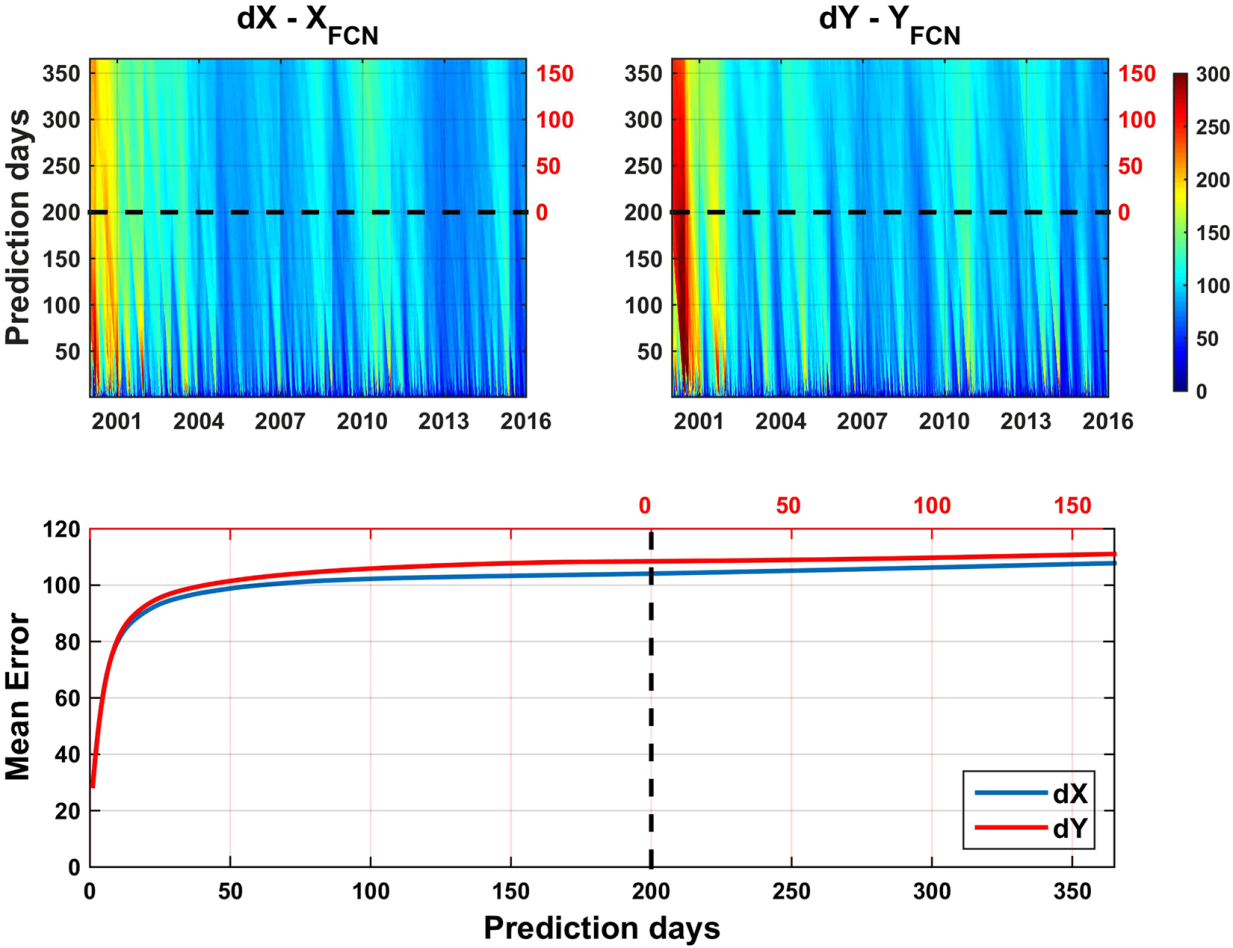
RMS of the differences (top), along with the mean prediction errors (bottom) between the observed CPO taken from IERS 08 C04 series and the corresponding daily CPO predictions. Black and red axes labeling colors as in Fig. 3. Sampling interval: 1 day. Units: µas.

Focusing on the global analysis summarized in Fig. 5 (analogous to Fig. 3), similar patterns and behavior become obvious from one-day to 365-days forecast over the whole time frame: 2000.0–2016.0. On the long-term, the statistical analysis of the whole period reveals that the errors are quasi flat over a wide time span of about ±150 days (around the 200 days half window length). Consequently, the proposed prediction method provides practically constant accuracy over time in 2000.0–2016.0 over a wide region around the center of each FCN fitting window when is taken as initial day for starting predictions.

On the basis of these results, we demonstrated how important the FCN model is for CPO prediction. The ability to make accurate and trustable predictions critically depends on the quality of this model. Besides, the use of high temporal resolution FCN models, i.e. small sliding window length and minimal displacement between subsequent fits, could make the method more sensitive to geophysical changes, e.g. sparse sudden events, helping to improve the forecast. Therefore, the promising results do not only depend on the simple approach which keep the latest amplitude and phase constant for the FCN prediction, but mainly on the complex estimation of suitable and reliable FCN models, as was recently demonstrated in certain articles^47,51^.

### Latency

Regarding the potential application of the method to provide operational predictions, we have to account for the latency associated with the availability of VLBI CPO results and the updating of the FCN model. The current system for the CPO prediction has certain particularities. Whilst the prediction of ERP and UT1-UTC starts the next day that Bulletin A is sent out by the IERS RS/PC, the “prediction” of the CPO starts at a past date earlier than its release, imposed by the availability of the individual and IERS combined VLBI solutions. For instance, the Bulletin released on May 10 2018 (MJD 58248) includes CPO predictions from MJD 58219 (April 24) to 58232, a time interval in which VLBI observations have been performed but their final analysis not yet completed. The term latency is used here with that particular meaning when applied to CPO and its value is around three to four weeks. According to Fig. 5, the deviations between our predictions and observations exhibit the lowest errors in the first three weeks. Therefore, the current latency of the C04 solutions implies that we cannot take advantage of the good capabilities of our method for ultra-short term prediction, understood as prediction for days in the future, nor in the past. Nevertheless, the behavior shown in the lower Fig. 5 is hardly affected for a few weeks latency due to the very slow growth of the mean deviation error.

### Statistical analysis of the residuals and comparisons

Finally, the accuracy of our CPO predictions when compared with the final CPO from IERS 08 C04 series has been tested and presented in Table 1, similar to Table 5 of the IERS Annual Report 2016 (AR2016)^16^. Accuracy is understood in this context as the ability of our predictions to reproduce the basic C04 CPO series, measured in terms of the RMS of deviations between predicted and observed values. Those RMS do not correspond to actual accuracies of predictions referred to the (unknown) actual CPO series, although of course we could add to them the estimates of the reported CPO errors in order to have an upper bound of the errors against “exact” CPO values. For each component (dX, dY), the column headed “AR2016” displays the values of the accuracy shown in that AR2016^16^, which are tabulated only up to 40 days forward. The next four columns for each CPO display the accuracies obtained with different delays or latency in the calculation or application of our procedure. An ideal case with no latency is included to give a better idea of the future possibilities of the method, since we expect that the present/future VLBI technique improvements will make feasible the obtaining of near real time solutions both for VLBI and FCN models. The next columns correspond to latencies of three weeks (comparable to the actual one in the current IERS CPO predictions), 60 days (easier to be implemented at short- to mid-terms) and 200 days. We have associated to the AR2016 column a latency of three to four weeks according to the former sub-section, although the meaning of latency here is quite different to that in the other columns, since it refers rather to the publication date of Bulletin A. Let us remark that the official predictions released by the IERS RS/PC do not rely on previous determinations of values unlike ours, which are based on previous CPO values determined and released by the IERS EOC. Therefore, the values for a prediction interval of 0 days of the aforesaid Table 5 correspond to the errors due to the systematic differences between the input to the prediction algorithm, which are the Rapid Service combination estimates, and the 08 C04 post processed values. Thus, this row of Table 5 may reflect the size of the systematic differences between the 08 C04 values and the Rapid Service combination values rather than the quality of the prediction process. This points out the importance of the input data accuracy to the prediction accuracy.

**Table 1:** RMS of the differences between the CPO prediction series produced by the daily solutions provided in the IERS Annual Report 2016 and the prediction series estimated in this study for different latencies, always with respect to the IERS 08 C04 series.

Days in Future	dX (µas)					dY (µas)				
	AR2016	This study				AR2016	This study			
	3–4 weeks	Latency				3–4 weeks	Latency			
		0 (ideal)	3 weeks	60 days	200 days		0 (ideal)	3 weeks	60 days	200 days
0	120	29,6	72,6	80,2	89,3	100	27,6	73,3	77,9	81,5
1	120	39,5	73,0	80,4	89,3	100	36,7	73,6	77,9	81,5
5	120	58,9	74,3	81,0	89,3	100	55,0	74,6	78,1	81,5
10	120	67,1	75,5	81,7	89,3	110	65,3	75,3	78,5	81,6
20	130	72,7	77,3	82,7	89,1	130	73,3	76,4	79,3	81,7
40	140	77,3	80,4	84,5	88,3	160	76,4	77,9	79,8	82,0
50	—	78,9	81,8	85,3	88,0	—	77,3	78,6	80,2	82,3
75	—	82,3	84,1	87,3	87,6	—	79,0	79,7	81,0	82,9
100	—	84,5	86,2	88,2	87,5	—	79,8	80,6	81,2	83,5
150	—	87,9	88,6	89,3	88,5	—	81,1	81,3	81,6	85,1

The new method (“This study”) gives smaller RMS than the official product (“AR2016”) in all “Latency” cases. According with the former remarks, it is difficult to estimate what part of the quantitative improvement in the predictions may be attributed to the prediction algorithms or to the effect of the systematic differences in the input series of data; clarifying that would require a further specific investigation. For better illustrating the gain in the repeatability of CPO determinations for each prediction procedure, we have displayed on Table 2 the percentage RMS decreasing of each procedure with respect to the official product reported in Table 5 of AR2016 (p. 105), arranged similarly than in Table 1. For dX the reduction range is 26–75%, with an average of 39.5% in the three weeks latency column. For dY the respective range and average are 19–72% and 33.8%.

**Table 2:** Reduction of the RMS for each cell in Table 1, expressed in percentage with respect to the corresponding AR2016 values.

Days in Future	dX					dY				
	AR2016 3–4 weeks	This study				AR2016 3–4 weeks	This study			
		Latency					Latency			
		0 (ideal)	3 weeks	60 days	200 days		0 (ideal)	3 weeks	60 days	200 days
0	0	−75,3	−39,5	−33,2	−25,6	0	−72,4	−26,7	−22,1	−18,5
1	0	−67,1	−39,2	−33,0	−25,6	0	−63,3	−26,4	−22,1	−18,5
5	0	−50,9	−38,1	−32,5	−25,6	0	−45,0	−25,4	−21,9	−18,5
10	0	−44,1	−37,1	−31,9	−25,6	0	−40,6	−31,5	−28,6	−25,8
20	0	−44,1	−40,5	−36,4	−31,5	0	−43,6	−41,2	−39,0	−37,2
40	0	−44,z8	−42,6	−39,6	−36,9	0	−52,3	−51,3	−50,1	−48,8

Besides, it can be seen that the accuracy is almost stationary or increases much slower than in the first column. For the AR2016 case, the accuracy increases from 100 to 160 µas for dY component, i.e. a 60%, whereas the 3 weeks latency in the same projection (40 days in future) increases from 73.3 to 77.9 µas, i.e. only about 6%. It means that the deterioration is ten times smaller, which allows us to extend the prediction time interval about four times, up to 150 days – and actually beyond. Consequently, we could say that the proposed method significantly improves both the stability and accuracy of the prediction of the IERS EOP 08 C04 CPO values.

## Discussion

In this work, we investigated the CPO prediction procedure built on adopting the FCN model B16^48^ to predict the contributions to CPO attributable to the FCN. The B16 model is empirical and fitted to VLBI data using a sliding window length of 400 days, a displacement step size of one day and a constant period of −431.18 sidereal days. The model being determined from observations, we have to cope with a latency of 200-day in the determination of the B16 FCN amplitudes and phases. Besides, the CPO values are not provided in near real time after finishing a VLBI session, what produces an additional latency that can reach some weeks for the C04 solution since it requires a combination of solutions released by several IERS AC. Because of that we label the near real time or zero-day latency case in Table 1 as “ideal”, and select as better reference a latency of three weeks, taking into account the time needed to derive and release the final C04 solution. It is expected that the latency will keep its decreasing trend in the near future, but at present it would be unrealistic consider latencies of e.g. one week, which would benefit the performance of our method, indeed. Despite all those obstacles, and not forgetting the issue of the systematic error mentioned above, our results clearly demonstrate that it is possible to decrease the RMS of the deviations of the official CPO prediction with respect to the conventional CPO determinations at a level of 40–50% in the case of a three weeks latency and a 40 days long prediction time span. The gain is understood and measured in terms of the RMS of the deviations between a CPO prediction and the corresponding determined daily values taken from a reference time series, EOP 08 C04 in our study. Those “observed” values are taken as exact to estimate the goodness of the predicted CPO series as made in other assessments of prediction accuracy^50,53^. Actual values and accuracies of CPO are of course out of reach, although we could consider figures between 40 and 90 µas as plausible benchmarks to estimate the 1-σ accuracy of the CPO, according to the comments exposed in the introduction and the authors’ interpretation.

The accuracy of the predictions of both dX and dY, understood as explained before, is kept within the range 80–90 µas after 100 and 150 days whatever the latency time indicated in Table 1. That behaviour is comparable to that of the ZM2 method by Malkin^53^. ZM2 method is quite different from ours, since it does not focus on the FCN component but proceeds by calculating a Gaussian smoothing of each CPO series taken from the IVS solution, depending of certain smoothing parameter. ZM2 CPO predictions are derived from an autoregression fitting and their RMS after one-month when compared to the IVS series is about 75–100 µas depending on the smoothing parameter, and after 100 days it exceeds slightly 100 µas (see Fig. 3 in^53^). The error growth is almost uniform along that 100-day period. Lambert’s method allows FCN prediction with the limitation inherent to its low frequent updating, once a year. Like Malkin’s, it exhibits also an almost linear error growth, at a rate of 0.1325 µas/d along a 700 days time interval as reported in^50^ - about 48.4 µas/y. The error growth in our method shows a quite different pattern, visible in Fig. 5. For dX the mean rate along 40 days is 0.50 µas/d (in contrast to the 1.19 µas/d deduced from the reported RS/CP RMS values), but in the whole 150 days period the mean decreases till 0.04 µas/d. The behaviour of dY is better; the respective values are 0.12 and 0.03 µas/d after 40 and 150 days, while the AR2016 mean rate value is 1.50 µas/d at the 40^th^ day.

Summarizing the previous noteworthy features, we can conclude that accurate prediction of the FCN signal with respect to the B16 modelled FCN can be done based on its prior amplitudes with a mean error of about 30 µas/year, with a nearly linear trend (bottom Fig. 3). Besides, the RMS of the differences between the CPO produced by the IERS EOC, namely C04 series, and our predicted CPO based simply on a thorough FCN prediction over 2000.0–2016.0 exhibits an almost logarithmic-like growing that stays rather constant below 120 µas for more than a year (bottom Fig. 5).

The comparison of the current CPO prediction method with the one reported by the IERS RS/PC, shown in Tables 1 and 2, demonstrates that our empirical predictions attain a substantial reduction of the unexplained RMS deviations between predicted and observed values for both the dX and dY components despite accounting for a realistic data latency. Maybe part of that good behavior comes from the fact it is not affected by possible systematic differences between the RS/CP input for dX and dY and the 08 C04 combination values in 2016. The mean reduction in Table 2 for dX is 40%, and for dY 35%. After 40 days there are no reference values in the AR2016, but the RMS growth up to 150 days is less than 10 µas for dX and dY. Roughly speaking, the overall reduction is around 40% for any time span up to a year. Therefore, the tested empirical FCN model of high temporal resolution is promising to help to improve the CPO predictions, in the sense of making them closer to the conventional later observed CPO, from short to long-term, achieving an RMS error below 90 µas for each CPO for predictions up to 150-days. That error is clearly below the current operational uncertainty of the CPO predictions when compared to observations, which vary from 140 to about 160 µas in a much shorter 40-days interval. Despite the progress, the uncertainty we got is still insufficient to meet the present accuracy goals of global geodesy, set at 1 mm or 30 µas^58^. However, the reduction of the prediction uncertainty over longer time spans opens the way to the development or implementation of applications in near real time, with shorter latency. The applications that might benefit from improved CPO predictions are for example improved accurate prediction of orbits from single geodetic satellites to constellations of them or monitoring sea level variations with latency closer to real time.

Last, let us comment that our empirical method is not designed to consider the geophysical phenomena that excite the FCN. Therefore, new methods or a combination strategy of the existing methods could be investigated for improving the CPO prediction. In addition, the impact of mass redistribution and movement within the Earth system, such as solid Earth, atmosphere, ocean, hydrosphere, and cryosphere, on the EOP could be considered to get more insight into the reliability of the forecasting model^36^. Besides, stochastic methods that analyse and exploit the dependency structure between multivariate data could be applied to further study the yet unexplained EOP variations.
